# The risk of febrile neutropenia in patients with non-small-cell lung cancer treated with docetaxel: a systematic review and meta-analysis

**DOI:** 10.1038/sj.bjc.6604863

**Published:** 2009-02-03

**Authors:** A Wailoo, A Sutton, A Morgan

**Affiliations:** 1 Health Economics and Decision Science, School of Health and Related Research, University of Sheffield, Sheffield, UK; 2 Department of Health Sciences, University of Leicester, Leicester, UK; 3 Health Services Research, Sheffield, UK

**Keywords:** febrile neutropenia, docetaxel, meta-analysis, systematic review

## Abstract

We aimed to assess the incidence of febrile neutropenia in patients with non small cell lung cancer treated with docetaxel as second line chemotherapy by systematic review and meta-analysis of clinical studies. Published studies were retrieved and included if they considered docetaxel at the licensed dose after a previous chemotherapy regimen, and reported the proportion of patients getting FN. Meta-analysis was conducted to estimate the proportion of patients who experience one or more episodes of FN. The pooled, random effects meta-analysis estimate for the proportion of patients who experience one or more episodes of FN on docetaxel was 5.95% (95% CI 4.22–8.31) based on 13 studies, comprising 1609 patients. No significant differences were seen either between studies that permitted the use of prophylactic granulocyte colony-stimulating factors or between phase II and phase III trials.

Evidence from randomised controlled trials suggests that the incidence of FN with docetaxel is around 6% and therefore an important factor to consider in the choice of the chemotherapy regimen.

Chemotherapy-induced febrile neutropenia (FN) is a serious adverse event caused by cancer therapies that can have a significant impact on mortality, morbidity and health care costs. FN impacts on quality of life both directly and indirectly, because it may lead to serious and potentially fatal infections and also because chemotherapy treatments can only be delayed or reduced with potentially detrimental clinical consequences . In addition, patients almost always require hospitalisation and treatment with antibiotics.

To reduce the risk of FN, recombinant human granulocyte colony-stimulating factors (G-CSFs) or granulocyte–macrophage colony-stimulating factors (GM-CSFs) may be considered. These agents can be used to prevent FN in all patients receiving chemotherapy, but they are costly. Consequently, they have been recommended for use in treating and preventing FN only in high-risk patients (Aapro *et al*, 2006; NCCN, 2006; Smith *et al*, 2006), in whom the evidence for their effectiveness is strongest. Antibiotic prophylaxis is an alternative prevention strategy that has been considered in several studies, although concerns about widespread antibiotic resistance have limited the use of this strategy in practice (NCCN, 2006).

In considering alternative preventative strategies, the differential risks, costs and potential benefits must be weighed up. Therefore, it is essential to accurately identify the risk of FN associated with any particular chemotherapy regimen. The European Organisation for Research and Treatment of Cancer guidelines (Aapro *et al*, 2006) and American Society of Clinical Oncology guidelines (Smith *et al*, 2006) both state that where the risk of FN with a chemotherapy is in excess of 20%, G-CSF prophylaxis is recommended in that patient group, and that where the risk associated with a specific regimen is lower than 20% there should be consideration of other patient factors such as age and co-morbidities.

Yet information on the risk of FN is lacking in this area. In patients with non-small-cell lung cancer (NSCLC), three second-line treatments are currently licensed in Europe: pemetrexed (Alimta®, Eli Lilly and Company, Indianapolis, USA), docetaxel (Taxotere®, Sanofi-Aventis, Paris, France) and erlotinib (Tarceva®, Roche, Basel, Switzerland). Pemetrexed is rarely used in practice. In the UK National Health Service (NHS), pemetrexed is not recommended for use in this patient group by the National Institute for Health and Clinical Excellence (NICE, 2007a). On the other hand, docetaxel is widely used in practice and is recommended by NICE (NCCAC, 2005). A third treatment, erlotinib, is a newer oral treatment that has been the subject of a recent NICE appraisal. As a part of that appraisal, the cost effectiveness of erlotinib compared with docetaxel was estimated. As erlotinib does not have haematological toxicity, a crucially important component of the cost effectiveness estimate is the probability of FN in patients treated with docetaxel. Results are particularly sensitive to this probability (DSU, 2007) yet data on this parameter proved controversial (NICE, 2007b). The European Organisation for Research and Treatment of Cancer cite percentages of patients experiencing one or more episodes of FN of 26% for docetaxel in combination with carboplatin, and between 5 and 11% in combination with cisplastin. However, these estimates are based on only a small number of studies of varying quality, and as much of the evidence relates to first-line treatments, they may not be applicable for second-line NSCLC patients. The American Society of Clinical Oncology guidelines report that 12.7% of second-line NSCLC patients experienced FN but this is based on a single study (Hanna *et al*, 2004). We therefore conducted a systematic review and meta-analysis of docetaxel studies in NSCLC to identify the risk of FN, specifically for these patients.

## Methods

### Search strategy

A comprehensive search was undertaken to identify the literature on docetaxel use in NSCLC. The databases searched (for all years that were indexed) were Medline, Medline in Process, EMBASE and The Cochrane Library, including the Cochrane Database of Systematic Reviews, Cochrane Controlled Trials Register (CENTRAL), DARE, NHS EED and HTA databases. Searches were not restricted by language or publication type. The search strategy for EMBASE was modified to include additional terms around ‘Neutropenia’, as omitting these terms resulted in an unmanageable result set of limited specificity.

Earlier systematic reviews considering docetaxel were also considered so that a manual search of their reference lists could be conducted to ensure that all relevant studies had been identified. Studies that met the inclusion criteria mentioned above but were only published as abstracts or as conference presentations were not included in the review unless a full paper could be obtained that related to the abstract.

### Inclusion criteria

Studies were included if they assessed the use of docetaxel as monotherapy at the standard recommended dose (75 mg m^−2^ as a 1-h infusion every 3 weeks), in patients with NSCLC who had received one or more previous chemotherapy regimens for their disease and for which FN events were reported.

### Data extraction

The primary outcome of the review was the rate of one or more episodes of FN among patients receiving docetaxel. We also sought to extract information on the grade of neutropenia and the proportion of patients receiving either G-CSFs or antibiotics to prevent or treat FN in those studies. Additional information regarding the baseline characteristics of the patient populations who took part in the studies and description of survival outcomes and treatment duration were also extracted and were reported.

### Evidence synthesis

Meta-analysis was performed on the log odds scale and results were transformed back to the proportion scale for interpretation. Heterogeneity between the studies was assessed using the *I*^*2*^ statistic and where it was greater than 0, a random effect meta-analysis model was used in preference over a fixed effect one. Where heterogeneity existed covariates were included in the analysis in an attempt to explain the between-study heterogeneity.

## Results

A total of 950 studies were identified from the literature searches. Titles and abstracts were scanned for relevance and full copies of 40 studies were ordered. Of these, 13 studies were selected for inclusion, eight of which were phase III randomised controlled trials (RCTs) (Fossella *et al*, 2000; Shepherd *et al*, 2000; Gridelli *et al*, 2004; Hanna *et al*, 2004; Schuette *et al*, 2005; Camps *et al*, 2006; Chen *et al*, 2006; Ramlau *et al*, 2006) and five were phase II RCTs (Quoix *et al*, 2004; Gervais *et al*, 2005; Pectasides *et al*, 2005; Wachters *et al*, 2005; Cufer *et al*, 2006). The relevant data were contained in single trial arms, which consisted of a total of 1609 patients (see Table 1). The comparators to standard dose docetaxel included different doses of docetaxel, best supportive care or other active treatments (irinotecan, topotecan, vinorelbine, ifosfamide, gefitinib or pemetrexed).

All studies report a mean patient age of around 60 years with a range of between the mid-30s and mid-70s in most studies. All patients have advanced NSCLC with the majority having metastatic disease (stage IV). The majority of patients in the trials have, however, good performance status (0 or 1); that is, they are ambulatory and are able to carry out work of a light or sedentary nature with few restrictions.

Seven of the 13 studies included described some use of G-CSF in their study patients (Table 1). Only one of these reported prophylactic use of G-CSF in all study patients (Wachters *et al*, 2005). The remainder reported that a minority of study patients received G-CSF. Two studies reported data separately for the proportions of patients receiving G-CSF for prophylaxis: 1.4% (Hanna *et al*, 2004), and for treatment of FN: 17% (Chen *et al*, 2006) and 12.1% (Hanna *et al*, 2004). Two studies reported combined prophylactic and treatment use of G-CSF: 28% (Pectasides *et al*, 2005) and 8% (Ramlau *et al*, 2006). One study reported that G-CSFs were used in 7% of cycles either prophylactically or as treatment for FN (Fossella *et al*, 2000) and one study simply reported that G-CSFs were used at the physician's discretion without providing actual data (Schuette *et al*, 2005).

In all trials FN events are presented as the percentage of patients experiencing one or more episode of FN. The final column of Table 1 summarises these percentages for the arms of the studies, in which the standard doses of docetaxel were administered; that is, 75 mg m^−2^ given intravenously over 1 h every 3 weeks. The trials do not generally report the number of events per person and almost all studies combined the grades of FN together. In studies that did not prescribe G-CSFs, FN rates for patients treated with the recommended standard dose of docetaxel ranged from 1.8 to 7.8%. In studies that prescribed G-CSFs, FN rates ranged from 2.0 to 12.7%. None of the studies reported using prophylactic antibiotics.

### Meta-analysis

**Figure 1:**
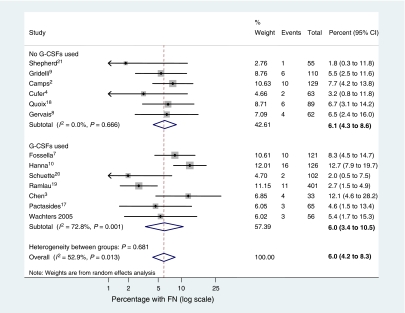
Forest plot for ln (odds) of experiencing FN on standard dose docetaxel (75 mg m^−2^ given intravenously once every 3 weeks).

Meta-analysis was conducted on the 13 study arms described in Table 1. Some numerator data for the percentage of patients with FN were not reported but these were derived directly using the denominators and percentages reported in the papers. Only one figure was equivocal – the numerator for Gridelli *et al* (2004) – as five or six events would provide a percentage rounding up or down to 5%. Six events were imputed in this instance. Hence the data used in the meta-analysis are provided in Figure 1.

Owing to between-study heterogeneity (*I*^*2*^=52.9%), a random effect meta-analysis was conducted. The pooled, random effects meta-analysis estimate for the proportion of patients who experience one or more episodes of FN on docetaxel is 5.95% (95% CI 4.22–8.31). We conducted a subgroup analysis comparing studies that reported any G-CSF use with those that did not report use of G-CSFs as well as an overall analysis (Figure 1). For studies in which no G-CSF use was reported, the proportion of patients experiencing FN was 6.07% (95% CI 4.27–8.63) compared with 6.01% (95% CI 3.36–8.32) for studies in which G-CSF use was reportedly permitted. Although effect sizes in both groups were almost identical, the majority of the observed heterogeneity was in the group, which had some G-CSF use (*I*^*2*^=72.8 and 0% for G-CSF and non-G-CSF groups, respectively).

A further subgroup analysis was carried out combining phase III and phase II trials separately. Pooled estimates were similar from both (phase II=5.54% (3.52–8.62) and phase III=6.08% (3.68–9.87)) with the majority of the heterogeneity being observed in the phase III studies (*I*^*2*^=70.6% for phase III and 0% for phase II).

## Discussion

The meta-analysis results show that the incidence of FN associated with docetaxel as second-line therapy in trial patients with advanced NSCLC is approximately 6%. Individual patients and clinicians will want to consider this information as one element of the risks and benefits associated with alternative available chemotherapy regimens, which must be weighed up. Similarly, in deciding whether FN prophylaxis, such as antibiotics or G-CSFs, are appropriate for patients receiving docetaxel, the risk associated with the chemotherapy regimen must be considered alongside the risk factors related to the individual patient (for example, age, performance status) and the underlying disease. In relation to current European and American guidelines on the use of G-CSFs, the incidence of FN with docetaxel in NSCLC is relatively low.

This information is also a critical factor in policy-level decisions, such as those made by NICE, when considering the effectiveness and cost-effectiveness of available alternatives. In instances in which the figures for FN calculated in this report are used in assessing the cost effectiveness of erlotinib compared with docetaxel, it is considered unlikely that erlotinib is a cost-effective treatment (NICE, 2008), although this conclusion is obviously dependent on many other factors, including the price of erlotinib.

It is important to note that in routine practice, FN rates might be different, as the clinical trial participants, although potentially more likely to have advanced disease, are often younger and have better performance status than those who do not choose to participate (Elting *et al*, 2006). In addition, the FN rates reported in this review are those associated with licensed use of docetaxel; that is, 75 mg m^−2^. FN rates associated with larger doses of docetaxel, for example, 100 mg m^−2^, tend to be higher: 12 *vs* 8% (Fossella *et al*, 2000) and 22.4 *vs* 1.8% (Shepherd *et al*, 2000), for 100 and 75 mg doses, respectively.

These estimates may be limited by the reporting of G-CSF use within the trials and the necessity to categorise studies somewhat crudely as either using G-CSFs or not. The analysis does not consider the proportion of patients being administered G-CSF within studies. It is perhaps surprising that no specific observational studies were identified for this review, although such studies would be appropriate to improve the evidence base and would also allow factors that can be obscured in multi-national trials to be addressed. For example, the degree of clinical experience in using docetaxel, as with any chemotherapy regimen, is likely to be an important factor in achieving good patient management of which low rates of FN would be considered an important element. For these reasons, future observational studies could add to this meta-analysis and allow health system-specific issues to be considered.

## Figures and Tables

**Table 1:** Characteristics of included studies

		**Characteristics of trial patients on docetaxel 75 mg m^−2^**				
**Study**	**Interventions**	**Age median (range)**	**Tumour stage *N* (%)**	**Performance status ECOG *n* (%)**	**G-CSF use**	**FN *n* (%)**
Camps *et al* (2006) Spain	Docetaxel 75 mg m^−2^ one infusion every 3 weeks, *n*=131, *vs* docetaxel 36 mg m^−2^ one infusion every week for 6 weeks followed by 2-week rest, *n*=128. Treatment continued until disease progression or unacceptable toxicity.	61 (36–80)	IIIB 19 (14.8) IV 107 (82.9)^a^	0 31 (24) 1 77 (59.7) 2 21 (16.3)		10/129 (7.8)
Chen *et al* (2006) Taipei	Docetaxel 35 mg m^−2^ i.v. infusion on days 1, 8 and 15 every 4 weeks, *n*=64, *vs* docetaxel, 40 mg m^−2^ i.v. on days 1 and 8 every 3 weeks, *n*=64, *vs* docetaxel, 75 mg m^−2^ every 3 weeks, *n*=33. Treatment continued until disease progression or unacceptable toxicity.	64 (34–83)	IIIB 3 (9.1) IV 30 (90.9)	0 0 1 13 (39.4) 2 20 (60.6)	Used as treatment for all patients with FN, *n*=4 (12.1%)	4/33 (12.1)
Cufer *et al* (2006) International	Gefitinib oral dose of 250 mg per day, *n*=68, *vs* i.v. docetaxel 75 mg m^−2^ per day on day 1 every 3 weeks, *n*=73. Treatment continued until disease progression or unacceptable toxicity.	59.5 (29–83)	IIIB not reported^b^ IV 41 (56.2)	0 11 (15.1) 1 41 (56.2) 2 21 (28.8)		2/63 (3.2)
Fossella *et al* (2000) USA	Docetaxel 100 mg m^−2^ one infusion every 3 weeks, *n*=125, *vs* docetaxel 75 mg m^−2^ one infusion every 3 weeks, *n*=125, *vs* vinorelbine on days 1,8 and 15 of every 3-week cycle or ifosfamide on days 1–3 of every 3-week cycle, *n*=123. Treatment continued after six cycles if condition satisfactory.	59 (not reported)	III not reported IV 113 (90)^c^	0 not reported^d^ 1 not reported 2 23 (18)	Prophylactic or therapeutic use in 7% of patients	NR/121 (8)
Gervais *et al* (2005) France	Docetaxel 75 mg m^−2^ one infusion every 3 weeks, *n*=62, *vs* docetaxel 40 mg m^−2^ weekly for 6 weeks and a 2-week rest, *n*=63. Treatment continued until disease progression or unacceptable toxicity.	59 (37–72.5)	IIIB 21 (34) IV 41 (66)	0 9 (15) 1 40 (65) 2 13 (21)		4/62 (6.5)
Gridelli *et al* (2004) Italy	Docetaxel 75 mg m^−2^ one infusion every 3 weeks for six cycles, *n*=110, *vs* docetaxel 33.3 mg m^−2^ on days 1, 8, 15, 22, 29 and 36 every 8 weeks (6 weeks of treatment followed by 2 weeks of rest) for two cycles, *n*=110. Further therapy was discretional.	62 (26–74)	IIIB 21 (19) IV 89 (81)	0 35 (32) 1 58 (53) 2 17 (15)		NR/110 (5)
Hanna *et al* (2004) USA	Pemetrexed 500 mg m^−2^ as an infusion, *n*=283, *vs* docetaxel 75 mg m^−2^ as an infusion, *n*=288, on day 1 of a 21-day cycle. Treatment continued until disease progression or unacceptable toxicity.	57 (28–87)	III not reported IV 215 (74.7)^e^	0 or 1 252 (87.6) 2 36 (12.4)	Prophylactic use in 4 (1.4%) patients and as treatment in 49 (17%) patients	NR/126 (12.7)
Pectasides *et al* (2005) Greece	Docetaxel 75 mg m^−2^ one infusion every 3 weeks, *n*=65, *vs* docetaxel 30 mg m^−2^ (1-h infusion) and irinotecan 60 mg m^−2^ (90-min infusion) on days 1 and 8, both administered every 3 weeks, *n*=65. Treatment continued until disease progression or unacceptable toxicity.	59 (38–76)	Disease stage not reported	0 20 (31) 1 37 (57) 2 8 (12)	Therapeutic or prophylactic use in 18 (28%) patients	3/65 (4.6)
Quoix *et al* (2004) France	Docetaxel 100 mg m^−2^ one infusion every 3 weeks, *n*=89, *vs* docetaxel 75 mg m^−2^ one infusion every 3 weeks, *n*=93. Study planned for six cycles and further treatment could be given at physician's discretion.	59.0 (36.0–74.2)	I 5 (5.4) II 2 (2.1) III 8 (8.6) IIIB 20 (21.5) IV 58 (62.4)	0 16 (17.2) 1 53 (57.0) 2 24 (25.8)		6/89 (6.7)
Ramlau *et al* (2006) International	Oral topotecan 2.3 mg m^−2^ per day on days 1–5, *n*=414, *vs* i.v. docetaxel 75 mg m^−2^ per day on day 1 every 3 weeks, *n*=415, for at least four cycles. Additional cycles permitted.	58.7 (24–82)	III 117 (28) IV 298 (72)	0 76 (18) 1 273 (66) 2 65 (16) 4 1 (<1)	Administered to 30 (8%) patients	11/401 (2.7)
Schuette *et al* (2005) Germany	Docetaxel 75 mg m^−2^ one infusion every 3 weeks, *n*=103, *vs* docetaxel 35 mg m^−2^ on days 1, 8 and 15 of a 28-day cycle, *n*=105. Patients to receive a maximum of eight cycles of their regime.	63 (42–80)	IIIB not reported IV not reported^f^	0 34 (33.0) 1 55 (53.4) 2 12 (11.7) Non-assessable 2 (1.9)	Used at physician's discretion. No data on actual use reported	2/102 (2.0)
Shepherd *et al* (2000) Canada	Docetaxel 100 mg m^−2^ one infusion every 3 weeks, *n*=49, *vs* docetaxel 75 mg m^−2^ one infusion every 3 weeks, *n*=55, *vs* best supportive care, *n*=100. Treatment continued until disease progression or unacceptable toxicity.	61 (37–73)	IIIA/B 15 (27.3) IV 40 (72.7)	0 13 (23.6) 1 28 (50.9) 2 14 (25.5)		1/55 (1.8)
Wachters *et al* (2005) Netherlands	Docetaxel 75 mg m^−2^ one infusion every 3 weeks, *n*=56, *vs* docetaxel 60 mg m−^*2*^ plus irinotecan 200 mg m−^*2*^ as one infusion every 3 weeks, *n*=52. Treatment given for a maximum of five cycles.	59 (36–78)	IIIB 14 (25) IV 42 (75)	0 10 (18) 1 39 (70) 2 7 (13)	Lenograstim administered to all patients on days 2–12	3/56 (5.4)

ECOG=Eastern Cooperative Oncology Group; FN=febrile neutropenia; G-CSF=granulocyte colony-stimulating factor.

a The study does not report disease stage for three patients.

b All patients had stage IIIB/IV disease.

c All patients had locally advanced or metastatic NSCLC.

d Most patients had performance status 0 or 1.

e All patients were stage III or IV.

f The study included patients with stage IIIB or IV disease.

## Appendix 1

### Search terms

A comprehensive search was undertaken to identify literature on docetaxel use in Lung cancer. The search strategy for EMBASE was modified to include additional terms around ‘Neutropenia’ as omitting these terms resulted in an unmanageable result set of limited specificity. Searches were not restricted by language, publication date or publication type. Databases searched were Medline, Medline in Process, EMBASE and The Cochrane Library, including the Cochrane Database of Systematic Reviews, Cochrane Controlled Trials Register (CENTRAL), DARE, NHS EED and HTA databases.

#### Search strategy for Medline and Cochrane Databases

1. Carcinoma, Non-Small-Cell Lung/
2. Lung neoplasms/
3. docetax?l.tw.
4. docetax?l.rn.
5. docetax?l.nm.
6. taxotere.mp.
7. 1 or 2
8. or/3–6
9. 7 and 8

#### Search strategy for EMBASE

1. Lung non Small Cell Cancer/
2. Lung cancer/
3. docetax?l.tw.
4. docetax?l.rn.
5. docetax?l.nm.
6. taxotere.mp.
7. 1 or 2
8. or/3–6
9. 7 and 8
10. neutropen$.tw.
11. neutropaen$.tw.
12. 9 and 12
